# A CMOS-MEMS BEOL 2-axis Lorentz-Force Magnetometer with Device-Level Offset Cancellation

**DOI:** 10.3390/s20205899

**Published:** 2020-10-19

**Authors:** Josep Maria Sánchez-Chiva, Juan Valle, Daniel Fernández, Jordi Madrenas

**Affiliations:** 1 Electronic Engineering Department, Universitat Politècnica de Catalunya, Jordi Girona 1–3, 08034 Barcelona, Spain; juan.valle.fraga@gmail.com (J.V.); jordi.madrenas@upc.edu (J.M.); 2 Laboratoire de Recherche en Informatique (LIP6), Sorbonne Université, 4 place Jussieu, 75005 Paris, France; 3 Institut de Física d’Altes Energies (IFAE-BIST), Edifici Cn. Facultat Ciències Nord, Universitat Autònoma de Barcelona, 08193 Bellaterra (Barcelona), Spain; dfernandez@ifae.es

**Keywords:** MEMS, magnetic sensor, magnetometer, Lorentz-force, offset suppression, micromachined Resonator, micromechanical oscillator

## Abstract

Lorentz-force Microelectromechanical Systems (MEMS) magnetometers have been proposed as a replacement for magnetometers currently used in consumer electronics market. Being MEMS devices, they can be manufactured in the same die as accelerometers and gyroscopes, greatly reducing current solutions volume and costs. However, they still present low sensitivities and large offsets that hinder their performance. In this article, a 2-axis out-of-plane, lateral field sensing, CMOS-MEMS magnetometer designed using the Back-End-Of-Line (BEOL) metal and oxide layers of a standard CMOS (Complementary Metal–Oxide–Semiconductor) process is proposed. As a result, its integration in the same die area, side-by-side, not only with other MEMS devices, but with the readout electronics is possible. A shielding structure is proposed that cancels out the offset frequently reported in this kind of sensors. Full-wafer device characterization has been performed, which provides valuable information on device yield and performance. The proposed device has a minimum yield of 85.7% with a good uniformity of the resonance frequency $\bar{f_{r}}=56.8$ kHz, $\sigma_{f_{r}}=5.1$ kHz and quality factor $\bar{Q}=7.3$, $\sigma_{Q}=1.6$ at ambient pressure. Device sensitivity to magnetic field is 37.6fA·μT${}^{-1}$ at P=1130 Pa when driven with $I=1\;{\mathrm{mA}}_{\mathrm{pp}}$.

## 1. Introduction

In the last years, magnetometers have been introduced in a wide range of applications, increasing their demand and popularity [1]. Low end magnetometers in consumer electronic devices, with resolutions around some thousands of nanoTesla, are dominated by Hall sensors, devices based in the magnetoresistive effect (xMR), and Fluxgate sensors [2]. However, these solutions usually show large offsets and can not be integrated in the same die together with the electronics, requiring to stack multiple dies in a single package. On the other hand, Superconducting Quantum Interference Device (SQUID) magnetometers used in medical and research applications can detect fields below the nanoTesla level, but those sensors need extreme temperature conditions to operate conveniently, making them bulky and impossible to shrink [3].

Microelectromechanical Systems (MEMS) magnetometers based in the Lorentz-force have received significant attention by researchers due to their simplicity and flexibility. Because of the Lorentz-force sensing principle, the device sensitivity can be conveniently adjusted by changing the driving current. As a consequence, noise as low as 10nT$/\;\sqrt{H\;z}$, very close to SQUID counterparts, has been reported [4]. Moreover, being MEMS sensors, it would be feasible to batch manufacture them in the same die of MEMS accelerometers and gyroscopes, opening the door to the deployment of miniature sensor combos into the market.

Nevertheless, Lorentz-force MEMS magnetometers still face important bottlenecks that must be solved prior to their introduction into either commercial or medical applications. First, most devices reported in the literature require driving currents of various mA for the detection of Earth magnetic field and biasing voltages as high as 8 V [5,6]. As a consequence, integrated solutions would require complex and large area voltage boosting charge pumps. Moreover, high driving currents is an unacceptable requirement in nowadays power lowering trend. For this reason, low bias voltage and low current devices with good sensitivity must be developed. And second, MEMS magnetometers suffer from an important amount of offset, as detailed in Section 3.

In this article, a 2-axis MEMS magnetometer with a device level offset cancellation is presented. The device was designed using the metal layers available in the Back-End-Of-Line (BEOL) of a standard CMOS (Complementary Metal–Oxide–Semiconductor) process, opening the doors to a future co-integration, side-by-side, in the same die area with the electronics. Moreover, integration with CMOS-MEMS accelerometers and pressure sensors developed by the research group would be possible [7,8]. The design was done taking into account the need of achieving a good sensitivity with lower current and biasing voltage. Finally, this article discloses MEMS device performance data measured at wafer level. These results are then related to the BEOL metals curvature reported in the literature. Thus, this data may be useful not only for researchers, but also for industry designers that want realistic information on full-wafer.

This article is organized as follows. Section 2 describes the working principle of Lorentz-force resonant MEMS magnetometers, while Section 3 shows an analysis of the different offset sources that arise in such devices. The proposed device is described in Section 4, and the experimental results are depicted in Section 5. Finally, Section 6 discusses the results, while Section 7 describes the conclusions of the work.

## 2. Device Working Principle

**Figure 1:**
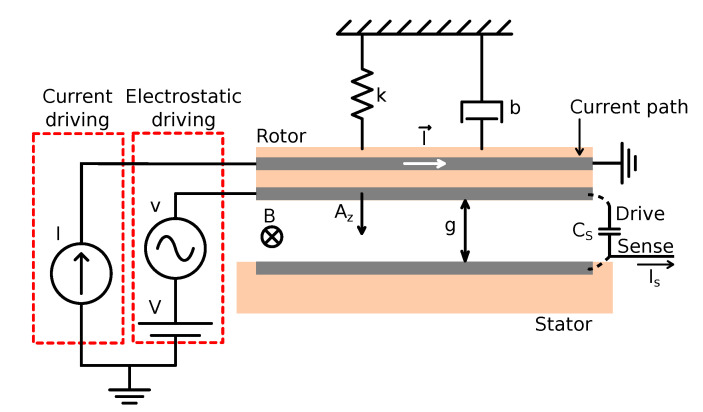
MEMS electromechanical model simplified diagram.

The proposed magnetometer is based on the Lorentz-force equation for current, which describes the force that a current carrying conductor suffers under the presence of a magnetic field,

(1) $$\vec{F_{L}}=I\vec{L}\times \vec{B}$$

where *I* is the injected current, $\vec{L}$ is the equivalent device length, and $\vec{B}$ is the magnetic field. When this current flows through a MEMS rotor, the resulting force generates an orthogonal displacement of the movable structure, as shown in the simplified MEMS diagram with the device electromechanical model in Figure 1. Moreover, if the injected current is at the mechanical resonance frequency, so does the Lorentz-force, resulting in an equivalent gap change $A_{z}$ described by [9,10]. In addition, Lorentz-force magnetometers are also usually driven electrostatically in order to sustain device oscillation when there is no magnetic field present. As a result, both the current and voltage driving generate a plate displacement defined as

(2) $$A_{z}\left(f_{r}\right)=\frac{Q}{k}\left(F_{L}+F_{E}\right)\approx \frac{Q}{k}\left(I\cdot L\cdot B+Vv\frac{C_{s}}{g}\right)$$

where *Q* is the device quality factor, *k* is the spring stiffness, $F_{E}$ is the electrostatic force, *V* is the device dc biasing voltage, *v* is the ac voltage or electrostatic driving, $C_{s}$ is the device capacitance and *g* is the nominal gap. The effect of electrostatic driving has been included because, being a resonant device, it is of utmost importance to track the resonance frequency, which is usually done by placing the device in a self-sustained oscillation loop [9,10,11,12,13,14]. When there is no magnetic field, some amount of electrostatic driving is required in order to keep the loop working correctly at resonance [14]. In this situation, when the current and electrostatic drivings are in phase, the device works in amplitude modulation (AM), and the equivalent gap change in Equation (2) generates a change of the device capacitance that follows.

(3) $$\Delta C_{s}=\varepsilon_{r}\varepsilon_{0}A\left(\frac{1}{g}-\frac{1}{g-A_{z}}\right)$$

where $\varepsilon_{r}=1$ is the air relative permittivity, $\varepsilon_{0}\approx 8.854\cdot 10^{-12}F/m$ is the absolute permittivity, and *A* is the plate area.

When the MEMS sensor has some amount of biasing voltage *V* and is driven with an ac current *I*, the gap variation generates a charge movement defined by $i_{s}=dq\left(t\right)/dt$

(4) $$i_{s}\approx \frac{\varepsilon_{r}\varepsilon_{0}AQV\omega_{r}}{g^{2}k}\left(\;\underset{\mathrm{Lorentz}}{\underbrace{I\cdot L\cdot B}}\;+\;\underset{\mathrm{Electrostatic}}{\underbrace{Vv\frac{C_{s}}{g}}}\;\right)$$

where $\omega_{r}$ is the device resonance angular frequency. The resulting device sensitivity is

(5) $$S_{i_{s}}\left(B\right)=\frac{\partial i_{s}}{\partial B}=\frac{\varepsilon_{r}\varepsilon_{0}AQV\omega_{r}IL}{g^{2}k}$$

Where it can be seen that sensitivity may be boosted by making the device area larger (increasing *A* and *L*) and softer (reducing *k*). Doing so, may reduce the need to increase the biasing voltage *V* and driving current *I*.

Device characterization as a function of the output current for a given magnetic field is very useful when a charge sensitive amplifier is used for the readout. However, given that the proposed device is characterized with an impedance analyser, a different approach must be used.

### 2.1. Device Characterization

**Figure 2:**
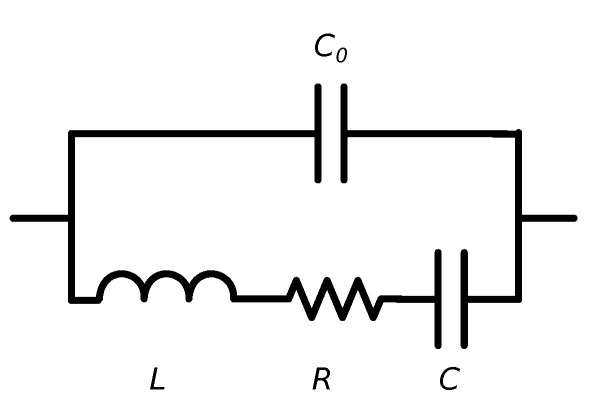
Second order RLC MEMS model used to fit the data.

MEMS measurements using the impedance analyser usually consist in measuring the device conductance (*G*) and susceptance (*B*) (or equivalent parameters), the real and imaginary parts of admittance respectively. Doing so, it is possible to extract the MEMS mechanical parameters by using the second order series RLC resonator model, shown in Figure 2. The admittance of the model is

(6) $$Y=\underset{\mathrm{Conductance},\;G}{\underbrace{\frac{R}{R^{2}+{\left(\omega L-\frac{1}{\omega C}\right)}^{2}}}}\;G+j\underset{\mathrm{Susceptance},\;B}{\underbrace{\left(\omega C_{0}+\frac{\omega L-\frac{1}{\omega C}}{R^{2}+{\left(\omega L-\frac{1}{\omega C}\right)}^{2}}\right)}}\;B$$

where *R*, *L*, and *C* are the MEMS equivalent resonator components, $C_{0}$ is the feedforward MEMS capacitance, and ω=2πf is the angular frequency. A Python script has been coded that fits the measurement data to the *G* model in Equation (6) and extracts the resonator RLC parameters using the least-squares minimization method. Then, MEMS resonance frequency $f_{r}$, and quality factor *Q* can be extracted with

(7) $$f_{r}=\frac{1}{2\pi }\sqrt{\frac{1}{LC}}$$

(8) $$Q=\frac{1}{R}\sqrt{\frac{L}{C}}$$

At resonance, the *L* and *C* terms cancel each other out and, as a result, the conductance term in Equation (6) is equal to $G(f_{r})=1/R$. Given that device output current expression in Equation (4) is an approximation only valid at resonance frequency, it can be related to the conductance with

(9) $$G\left(f_{r}\right)=\frac{i_{s}}{v}=\frac{\varepsilon_{r}\varepsilon_{0}AQV\omega_{r}}{g^{2}kv}\left(I\cdot L\cdot B+Vv\frac{C_{s}}{g}\right)$$

whose sensitivity to magnetic field can be derived as

(10) $$S_{G}\left(B\right)=\frac{\partial G(f_{r})}{\partial B}=\frac{\varepsilon_{r}\varepsilon_{0}AQV\omega_{r}IL}{g^{2}kv}$$

## 3. Offset Problems in Lorentz-Force Magnetometers: State of the Art

Lorentz-force MEMS magnetometers have numerous offset sources that limit their performance. The importance of these sources is strongly related to each device characteristics and topology. Nevertheless, they can be divided into two main categories: offset arising from voltage and offset arising from current drivings. In the following subsection, the offset sources related to each type of driving are identified and described.

### 3.1. Offset Arising from Voltage Driving

Applying an ac voltage driving to the device generates two different offset sources: MEMS plate actuation and signal feedthrough.

1. Plate actuation is a consequence of the resulting electrostatic force between the MEMS stator and rotor when applying an ac voltage driving to the device. Such electrostatic force generates a plate displacement that causes a gap change and a capacitance variation, as illustrated in Equations (2) and (3) respectively. In most AM Lorentz-force magnetometers, the electrostatic and current drivings are usually applied with the same frequency and phase. While plate displacement due to current driving (Lorentz-force) is desirable, displacement due to the electrostatic driving is an unwanted offset component. Such offset source is problematic as it reduces the dynamic range and has been demonstrated to worsen the long term instability [5,9].
2. Signal feedthrough is an offset that arises when interfacing the MEMS device with the readout electronics. If the sensor is placed in a capacitive half Wheatstone bridge readout circuit amplified with a fully-differential amplifier fed back with capacitors, the output signal $v_{o}$ as a function of the voltage driving $v_{i}$ is
  (11) $$v_{o}=v_{i}\frac{\Delta C}{C_{fb}}$$
  where $C_{fb}$ is the amplifier feedback capacitance and $\Delta C=C_{sensor}-C_{bridge}$ is the capacitance difference between the sensor and the bridge capacitances. In differential sensors with ideal matching ΔC=0 this feedthrough is completely compensated. The same happens with capacitive bridges ideally matched by using adjustable capacitors. However, MEMS capacitance drifts with temperature as a result of device springs coefficient change [15,16,17]. Moreover, MEMS capacitance may present variations due to fabrication and release non-idealities [18,19].

To avoid such offset sources, some works avoid the use of electrostatic driving: in [20,21] the sensor resonance frequency is tracked with resonators, but the sensing is performed at a frequency different from resonance, a technique called off-resonance driving. As a result, perfect resonance frequency tracking is not critical because the resulting sensitivity change at an offset frequency is minimized. Similarly, in [14], electrostatic driving is selectively enabled only when magnetic field is low enough to risk resonance unlocking. Although the resulting electrostatic ($F_{e}$) and Lorentz ($F_{L}$) forces are in quadrature, making it possible to remove the electrostatic driving offset with a synchronous demodulation, non-idealities due to this demodulation may arise, as well as capacitive bridge mismatch. To avoid that, in [9] complex modulation and demodulation schemes are proposed that successfully remove the electrostatic driving offset component. However, offset due to the current feedthrough to the sense electrode, as will be explained in the following subsection, can not be removed using this technique.

### 3.2. Offset Arising from Current Driving

There exist two types of offset sources related to the current driving: distributed electrostatic force along the current path, and capacitive coupling between the current path and sense node.

1. The fact that the resistance of the current carrying structure is not zero generates a voltage drop across the MEMS current driving path. This voltage drop between the MEMS current source and sink is translated into a distributed electrostatic force along the device that generates a plate displacement. Such issue is even worse in differential devices with current driven in series, as the resulting electrostatic force suffers an important mismatch. Some works [21,22,23] propose the adjustment of the voltage levels at these electrodes in order to compensate the mentioned electrostatic force imbalance. However, such solution requires manual adjustment, which is not feasible in mass production.
2. There exists a parasitic capacitive coupling between the current carrying path and the sense node, which results in a current feedthrough directly to the device output. In Ref. [14], a capacitive network between the current carrying path and the amplifier input is used to partly compensate this offset source, similar to the solution proposed in Ref. [24]. In Ref. [5] this source of offset is removed, together with other offset sources by using a complex modulation and demodulation strategy. Unfortunately, the proposed complex circuit still presents some amount of offset due to imperfections of the implemented circuitry.

### 3.3. Total Offset

Output current in Equation (4) only takes into account the current generated by Lorentz-force and electrostatic force plate displacements due to the device voltage driving. The former is the signal that carries magnetic field information, while the latter is the plate actuation offset source. However, it is possible to further detail the output current by adding the offset due to capacitive coupling and non-zero current path resistance

(12) $$i_{s}\approx \frac{\varepsilon_{r}\varepsilon_{0}AQV\omega_{r}}{g^{2}k}\left(I\cdot L\cdot B+Vv\frac{C_{s}}{g}+Vv_{c}\frac{C_{c}}{2g}\right)+\omega_{r}C_{c}v_{c}$$

where $C_{c}$ is the capacitive coupling between the current path and sense node, and $v_{c}=I\cdot R_{current}$ is the voltage applied to the current path, which is a function of the driving current *I* and the current path resistance $R_{current}$. Please note that capacitive coupling has been considered between current input and sense node, while plate displacement due to current driving has been considered to happen at the middle of the plate. Voltage feedthrough has not been included in Equation (12) because it appears only when the device is connected to a readout circuit, but it has already been depicted in Equation (11).

The offset removal proposed consists in shielding the current carrying path from the sense node, thus making $C_{c}=0$ F. For this reason, it aims to cancel the offset arising from capacitive coupling. Moreover, as the current path is shielded, so does the equivalent electrostatic force due to the current driving. Hence, as a result of the shielding, the device output current is finally the one depicted in Equation (4).

## 4. Proposed Device

**Figure 3:**
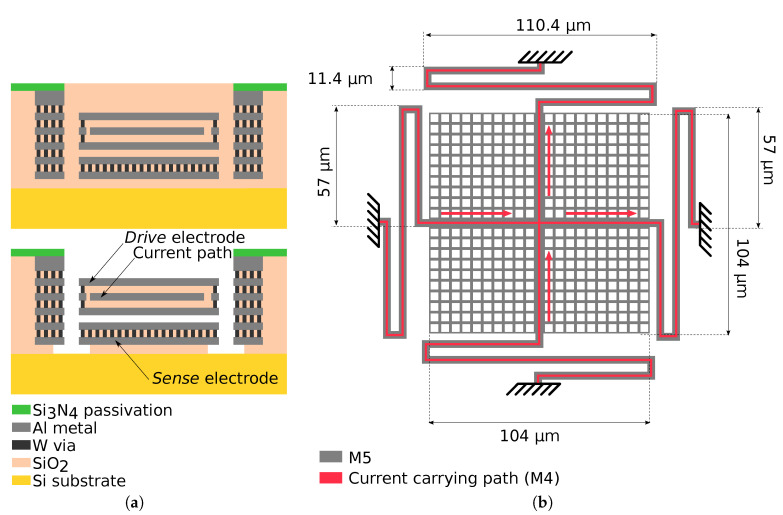
Device diagrams. In (**a**), simplified cross section diagram of the MEMS device manufactured using the BEOL layers of the CMOS process. On top, device at fab-out, below, after the release process with the different device parts depicted. In (**b**), device diagram with dimensions. The top-most metal layer (M5) is shown in grey, while the vertical and horizontal current carrying paths (M4) are shown in red. Red arrows show the different current flow directions that allow magnetic field measurement in two axis.

The proposed Lorentz-force MEMS magnetometer was designed and manufactured using the BEOL metal and oxide layers of a 6-metal 180 nm CMOS process. Then, the MEMS structure was wafer level released using vapour hydrofluoric acid (vHF), which etches away the oxide surrounding the MEMS, while keeping the structure metals and vias due to its high aluminium selectivity. The unmodified passivation layer provided by the foundry was used as a mask to protect the rest of the die from the acid, while a passivation window was open to allow the acid to attack only the MEMS areas. A simplified cross section diagram of the device and release process is shown in Figure 3a.

The device is a single-ended, parallel plate device that resonates in the out-of-plane resonance mode. The plate rotor consists of a stack of M3–M5 metals kept together with long vias, which provide both mechanical attachment and electrical connection between the different metal layers. Moreover, the use of various metal and oxide layers stack reduces the plate curvature after oxide release [19,25,26,27]. In order to allow the release agent to penetrate the MEMS structure and release the oxide between the rotor and the stator, the MEMS plate has 3.56μm×3.56μm openings uniformly distributed across the plate. Total plate dimensions are 104μm×104μm. A diagram of the MEMS device with its dimensions is provided in Figure 3b. Inside the plate, with M4, two 2.1μm wide metal tracks cross the plate, from left to right and top to bottom, in a cross-like shape providing a low-resistance path to the driving current. These tracks cross one to each other in the middle of the plate, but they are electrically insulated from the rest of M3–M5 rotor stack with unreleased SiO${}_{2}$, as vHF does not penetrate the structure due to the isolation provided by the metal and via stacks. The rest of the plate is connected to the *Drive* electrode. Doing so, the current carrying path is inside a Faraday cage-like structure that decouples the current from the *Sense* electrode. Due to its low impedance at circuit level, the *Drive* electrode is used as a shield. As a result, the two current related offset sources are avoided: first, there is no parasitic capacitance coupling between the current carrying path and the sense node and, second, these nodes do no generate an electrostatic force distribution.

**Figure 4:**
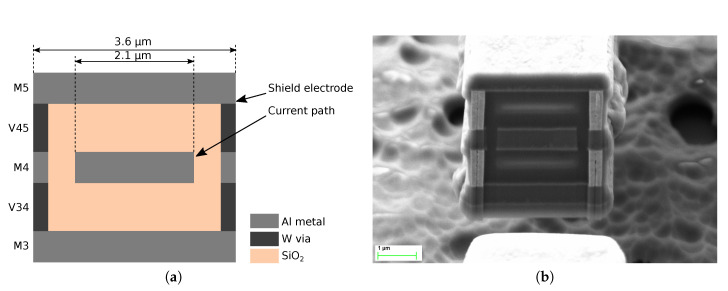
Device spring cross-section diagram (**a**) and image after a Focused Ion Beam (FIB) cut (**b**). Same as the rotor, the spring is made of a stack of M3–M5 metals and vias that surround the current carrying path at M4.

The device springs were designed with the same structure that the rest of the plate. The spring cross-section diagram as well as a Focused Ion Beam (FIB) cut image are depicted in Figure 4. As a consequence of the bulky cross-section of the springs, they were expected to have a relatively high stiffness.

The device stator is at M2, while M1 is used to connect the *Sense* electrode to the outside. The MEMS rotor is subjected to a frame that surrounds the entire device. This frame was designed as a stack of M1–M6 metals and long vias that stop the penetration of the vHF and protect the surrounding MEMS devices and electronics.

During the design, an important limitation was present: wafer level measurement setup required the samples to be tested at ambient pressure. In order to be able to measure devices resonance in such conditions, the final device springs had to be soft enough to provide a low restoring force easy to overcome electrostatically during the measurements. Moreover, soft springs also boost sensitivity, as deduced in Equations (5) and (10). For this reason, long springs were designed (110.4μm between meanders) in order to have a low stiffness coefficient. As a result of this design decision, the resonance frequency was lowered. Even though this fact simplified the device measurements and increased sensitivity, it must be taken into account that very low resonance frequencies should be avoided in order to minimize the presence of flicker noise in the amplifying stage to maximize the Signal-to-Noise-Ratio (SNR).

Finally, distance between samples across the wafer is around 19.5mm, and the proposed device is evenly replicated 56 times across a 8 inch wafer.

## 5. Experimental Results

In this section, the experimental measurements results are reported. However, in order to characterize the proposed device, different measurements were performed, each one requiring a specific experimental setup. For this reason, in the first part of the section, the various setups used are described.

### 5.1. Measurement Setups

#### 5.1.1. Wafer Level Batch Measurements

In order to understand the variations of the different device parameters across the wafer, wafer level batch measurements were performed. These measurements were carried out with probes using a MPI TS2000 (MPI Corporation, Chu-pei, Taiwan) semi automatic probe machine. Using this setup and a Keysight 4990A (Keysight, Santa Rosa, CA, USA) impedance analyser, the following measurements were performed:

- *Drive* to *Sense* capacitance and capacitance variation when sweeping the biasing voltage (C-V).
- Resonance measurements at ambient pressure.
- Current driving path resistance.
- Current driving electrode to *Sense* node parasitic capacitive coupling.

#### 5.1.2. Packaged Sample Vacuum Measurements

**Figure 5:**
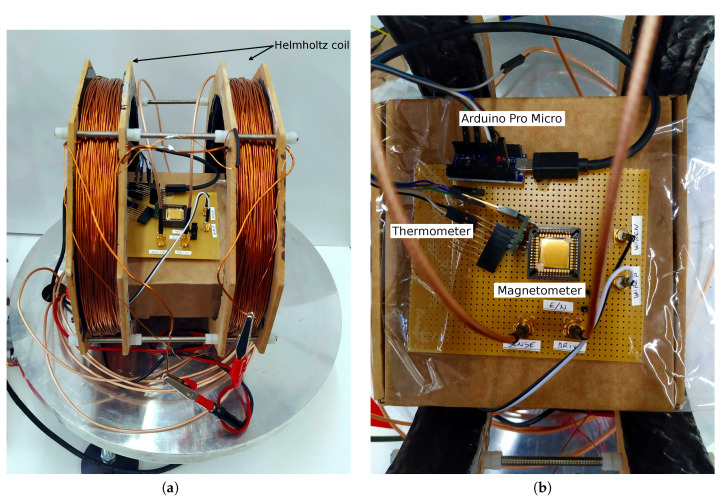
Images of the vacuum chamber setup. In (**a**), a general image of the setup right before placing the glass bell jar. The device chip is in the board socket, inside the Helmholtz coil. In (**b**), the board placed inside the coil is shown. The reference temperature sensor can be seen placed very close to the device, whose data is sent to a computer by an Arduino Pro Micro microcontroller board.

Next, the device needs to be placed in a vacuum environment in order to boost its Q and, as a result, its sensitivity to magnetic field. For such purpose, after performing all wafer level measurements, the wafer was diced and some devices were packaged and wirebonded to a JLCC44 (44 leads, J-Leaded Ceramic Chip Carrier) package. One sample was placed inside a vacuum chamber within a board socket with coaxial connectors connecting the *Drive* and *Sense* electrodes to the impedance analyser, as well as the current path electrodes connected to the outside of the chamber. Moreover, a single-axis, custom Helmholtz coil was also placed inside the vacuum chamber, with the board containing the sample in it. Images of the setup are shown in Figure 5. The pressure inside the chamber was measured with a CVM211 Stinger vacuum gauge (InstruTech. Inc., Longmont, CO, USA), while temperature inside the chamber was sensed with a LM95071 (Texas Instruments, Dallas, TX, USA) to be at 24 ${}^{\circ }\;C$ throughout the measurements.

**Figure 6:**
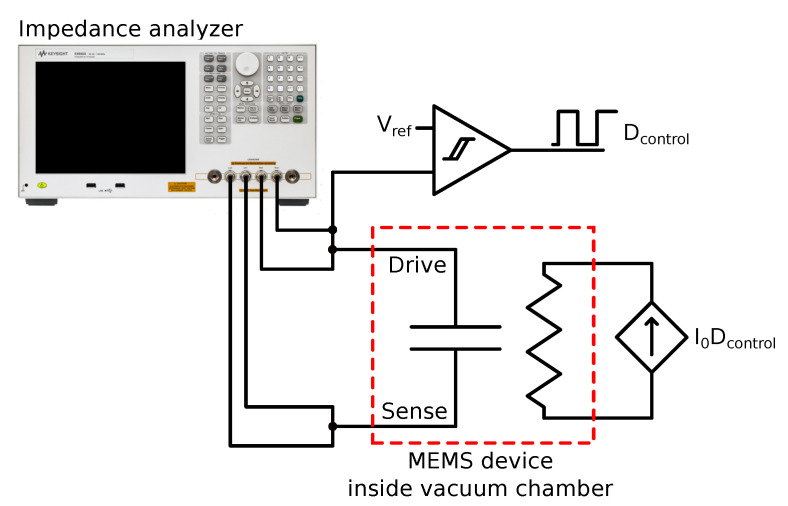
Schematic of the setup used to perform magnetic field sensitivity measurements. The impedance analyser performs 4-wire measurements. The analyser driving output is sampled and the signal sign $D_{control}$ is detected and used to drive an adjustable current source. As a result, the controlled current source output $I_{O}D_{control}$ is an ac current without dc component.

In order to allow measurements of the sensitivity to magnetic field, a current must flow through the device current carrying path with the same frequency and phase as the electrostatic driving. Doing so, the magnetometer works in AM, linearly modulating the plate vibration amplitude as a function of the magnetic field. To achieve that, the output of the impedance analyser is processed by the circuit shown in Figure 6. This circuit takes the output voltage of the impedance analyser, buffers it, and converts it into a square signal by means of a Schmitt trigger. The amplitude of such signal can be manually adjusted, allowing the selection of a given current driving. As a result, an amplitude adjustable current in phase with the impedance analyser electrostatic driving is created, which can be used to drive the device current path. More details on this setup are detailed in Ref. [28].

#### 5.1.3. Thermal Characterization

Device thermal characterization was performed by placing the board containing the sample inside an oven. After sufficient temperature stabilization time, resonance was measured with the impedance analyser. Measurements at ambient pressure were performed with temperature steps of 10 ${}^{\circ }$C from ambient temperature up to 100 ${}^{\circ }$C. Measurements above that value showed inconsistent Q and resonance frequency values likely due to tensions generated at the die as a result of socket thermal expansion.

### 5.2. Optical Analysis

**Figure 7:**
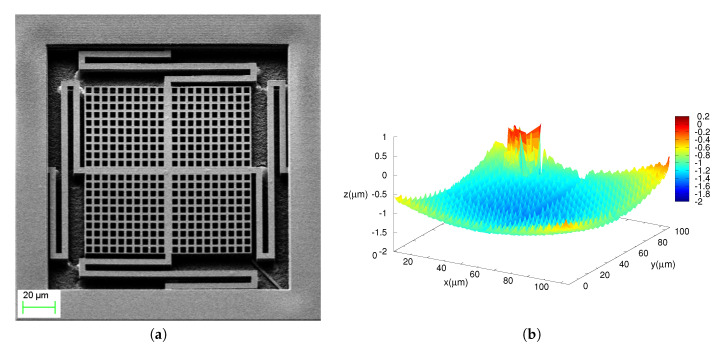
Device images: (**a**), a SEM microphotograph, (**b**) height profile of the device plate only, not including the springs nor the device frame. In the latter, the lump close to one corner is due to a tiny piece of dust on the plate.

A Scanning Electron Microscope (SEM) microphotograph of the manufactured device is shown in Figure 7a. Also, a confocal image of a device in the wafer periphery was taken with a Leika DCM 3D. The profile obtained is shown in Figure 7b. As it can be seen, the release of the MEMS results in a device with a concave plate curvature. Nevertheless, the stacked characteristics of the device result in a considerably flat device: the average curvature along the four springs is 0.361mm${}^{-1}$, while the maximum curvature of the plate, this is, from two diagonal corners is 0.343mm${}^{-1}$. Both values are within the range reported in Ref. [19] for the same via structure, and much lower than the curvature observed in single-metal structures in the same run.

### 5.3. Capacitance and C-V Variation Measurements

**Figure 8:**
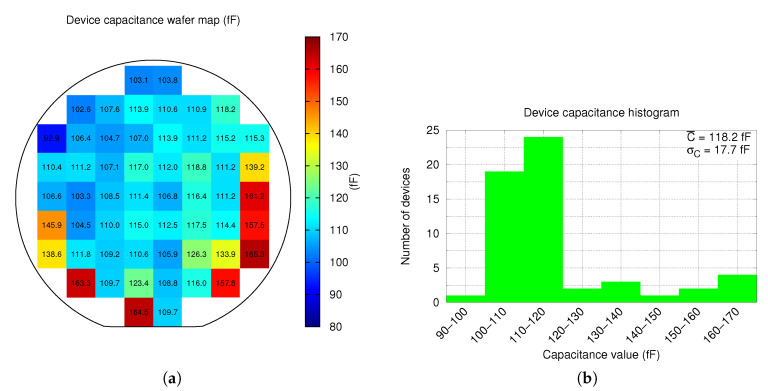
Wafer level measurements of device capacitance with 0V wafer distribution (**a**), and the histogram (**b**).

Device *Drive* to *Sense* electrodes capacitance with 0V biasing voltage measurements was performed with the impedance analyser in order to assess the uniformity of the device plate (*Drive*) position respect to the stator (*Sense*) across the entire wafer. This is an indirect way of assessing the device gap variability due to plate and spring curvatures. Figure 8a shows a map of capacitance measurements across all the wafer. In the figure, it can be seen that there is a good uniformity of capacitance values in the center of the wafer, being lower in the periphery upper half and higher in the periphery lower half. The histogram of the measurements is shown in Figure 8b, where it is possible to see, again, the good uniformity of the capacitance samples: an average capacitance $\bar{C}=118.2$ fF with a standard deviation $\sigma_{C}=17.7$ fF.

**Figure 9:**
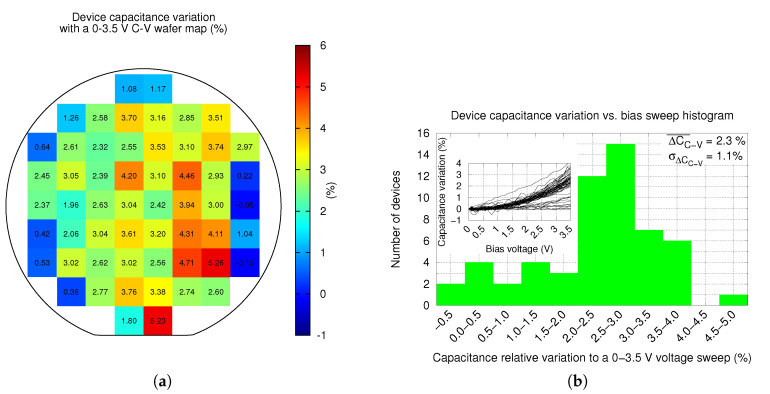
Wafer level measurements of device C-V normalized variation wafer distribution (**a**), and the histogram (**b**). All capacitance variation vs. biasing voltage sweeps are included in the inset of the latter.

Next, C-V measurements were performed as they provide important information of the device. First, it ensures that devices are correctly released and move as expected. And second, the plates and springs curvature is highly dependent on the metal residual stress, etching uniformity and etching temperature [18,19]. Hence, a uniform C-V variation ensures the correctness of these procedures. Figure 9a shows the normalized C-V variation distribution across the wafer, while Figure 9b shows the data histogram. In the former figure, it can be seen that the C-V variation is higher in the center of the wafer than in the periphery. Moreover, its uniformity is not as high, as it can be seen in the latter figure. In summary, the normalized C-V variation to a biasing voltage sweep of 3.5V is $\bar{\Delta C_{C-V}}=2.3\%$ with a standard deviation of $\sigma_{\Delta C_{C-V}}=1.1\%$.

### 5.4. Resonance Measurements at Ambient Pressure

**Figure 10:**
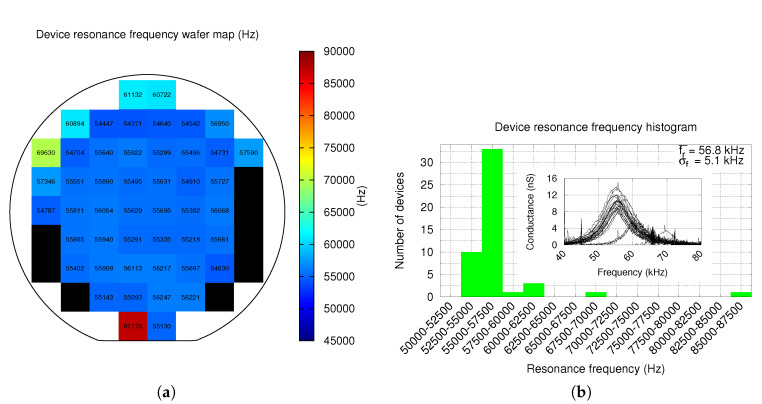
Wafer level measurements of device resonance frequency distribution map (**a**), and the histogram (**b**). All resonance measurements are included in the inset of the latter.

**Figure 11:**
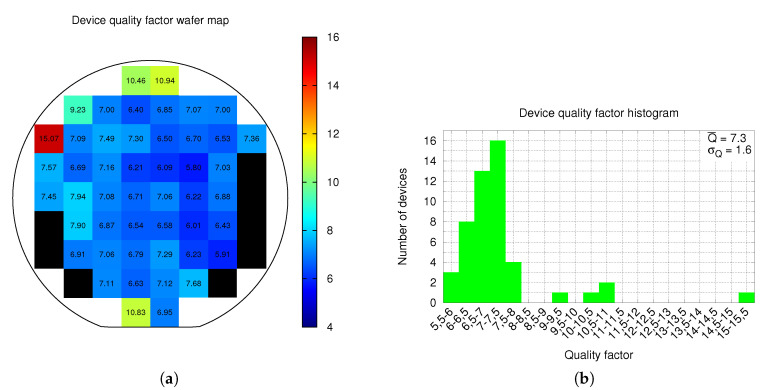
Wafer level device quality factor distribution map (**a**), and the histogram (**b**).

Resonance measurements in ambient pressure were performed for all devices with a 3 V biasing voltage and an ac driving of 100 mV. Resonance was found in 85.7% of the devices with an average value $\bar{f_{r}}=56.8$ kHz and a standard deviation $\sigma_{f_{r}}=5.1$ kHz. Resonance has a very good uniformity across all wafer, excepting the periphery, as depicted in Figure 10a. Measurements histogram is shown in Figure 10b. Resonance in the upper half of the wafer periphery is higher, as a consequence of the metals curvature distribution across the CMOS wafer as reported in Ref. [19]. Using the Python script mentioned in Section 2, the quality factor of each device was obtained from the conductance measurements. The quality factor distribution map is shown in Figure 11a, while the quality factor histogram is depicted in Figure 11b. The resulting average quality factor is $\bar{Q}=7.3$ with $\sigma_{Q}=1.6$, which can be considered a good Q factor for an ambient pressure measurement. Moreover, quality factor is, as expected from resonance results, very uniform across the inner part of the wafer as shown in Figure 11a.

Most devices of the wafer lower half periphery do not resonate. As a consequence, they are shown as black squares in Figure 10a and Figure 11a. The most plausible reason of this fact is that these devices have partially or totally collapsed due to hand contact as a result of an incorrect wafer manipulation. Various reasons support this assumption: first, these devices show a much larger capacitance in Figure 8a and a much lower (even negative) C-V variation in Figure 9a; and second, these devices are placed very close to where the wafer is hold. Thus, yield would significantly improve in a fully-automated production environment.

### 5.5. Current Driving to *Sense* Node Capacitive Coupling

The current driving path parasitic capacitance with the *Sense* node was also measured. The followed procedure was: first, the probes were placed over the device pads and the impedance analyser parasitic capacitance was calibrated; next, the probes for capacitance measurement were placed on the *Sense* electrode and one of the inputs of current driving, contacting the pads, while the probe of *Drive* electrode was connected to ground to provide the shielding. The measured parasitic capacitance coupling was, averaging all wafer samples, $\bar{C_{coupling}}=4.0$ fF. This value is very low, thus it can be considered that the shielding works as expected. The low, non-zero measured value is thought to be a consequence of either (1) instrument calibration resolution and (2) probes or wires uncompensated stray capacitance due to position change between calibration and probes making contact with the pads.

Finally, the minimum current path resistance measured is $R_{current}=46.3\;\Omega$, very close to the simulated value of 45Ω. The minimum measured value was considered as it is the one that provides the lower probe to pad contact resistance.

### 5.6. Device Sensitivity to Magnetic Field

**Figure 12:**
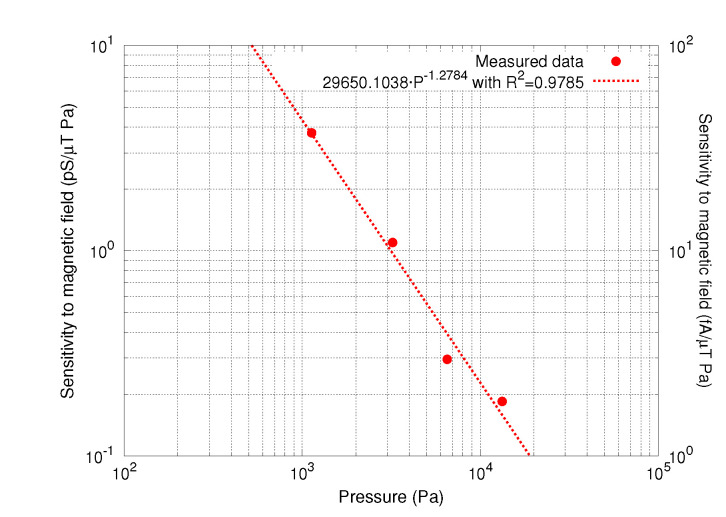
Device sensitivity to magnetic field, in pS(μT·Pa${)}^{-1}$, as a function of pressure. Each data point is obtained from the slope of conductivity when applying a magnetic field sweep between ±2mT.

As seen in Equation (10), device sensitivity to magnetic field is proportional to its *Q*, which is boosted at low pressure. For this reason, device sensitivity to magnetic field was measured at various pressure values in order to be able to assess the pressure at which the device may be packaged in a potential commercial solution. The measured device sensitivity to magnetic field as a function of the pressure is shown in Figure 12. Each sensitivity data point in the plot represents the conductance change as a function of the applied magnetic field in pS·(μT·Pa${)}^{-1}$ units (left vertical axis). Such slope is obtained from resonance measurements under the presence of a magnetic field sweep between ±2mT with 320μT steps for each pressure value. As expected, the device sensitivity boosts at lower pressures following a power law relationship of

(13) $$S_{G}\left(B\right)=29650.1\cdot P^{-1.2784}\;\frac{pS}{\mu T\cdot Pa}$$

for an electrostatic driving of v = 10 mV, a dc biasing of V = 1 V and a current of $I=1\;{\mathrm{mA}}_{\mathrm{pp}}$. Equation (13) is obtained from a power law fit. Unfortunately, at pressure lower than 600Pa, the device starts to enter into its nonlinear region with the mentioned electrostatic driving conditions. Lowering the ac driving voltage, linear measurements could be performed at lower pressures. Doing so, the packaging lower pressure limit as well as the maximum sensitivity can be further extended. Combining Equations (9) and (10), it is possible to see that device sensitivity in terms of output current can be expressed as

(14) $$S_{i_{s}}\left(B\right)=S_{G}\left(B\right)\cdot v$$

where *v* is the electrostatic driving voltage, a term that modulates the measured sensitivity in Equation (13), but that cancels out in Equation (14) with the *v* in the denominator of Equation (9). Sensitivity in terms of output current is also included in Figure 12 as seen in current units of fA·(μT·Pa${)}^{-1}$ (right vertical axis).

### 5.7. Device Sensitivity to Temperature

**Figure 13:**
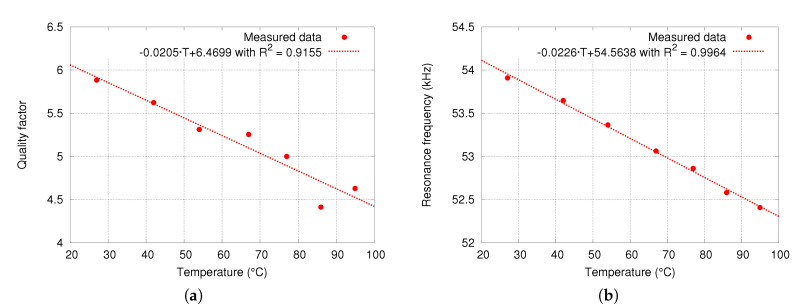
Measured quality factor variation (**a**) and resonance frequency (**b**) as a function of temperature at ambient pressure.

Device sensitivity to temperature change measurements provide an insight of the device quality factor variation, and its resonance frequency. As temperature increases, quality factor decreases, resulting in an equivalent reduction of device sensitivity to magnetic field that must be considered and compensated in order to obtain a constant sensitivity. Figure 13a quality factor variation as a function of temperature from room temperature up to around $100{\;}^{\circ }$C. Thermal coefficient of device quality factor is $-20.5\cdot 10^{-3}{\;}^{\circ }\;$C${}^{-1}$ ($-3163.3\;ppm\cdot^{\circ }\;$C${}^{-1}$).

Next, temperature also changes Young modulus of the spring materials, resulting in an equivalent spring coefficient change that is observed as a drift in the resonance frequency. Given that it is a resonant sensor, it is of utmost importance to track its resonance frequency. For this reason, it is important to know the extent of frequency variation in order to design the tracking circuit. Figure 13b shows the resonance frequency variation as a function of temperature. The resulting drift coefficient is −22.6Hz$\cdot^{\circ }\;$C${}^{-1}$ (−413.6ppm$\cdot^{\circ }$C${}^{-1}$).

### 5.8. Device Sensitivity to Pressure

**Figure 14:**
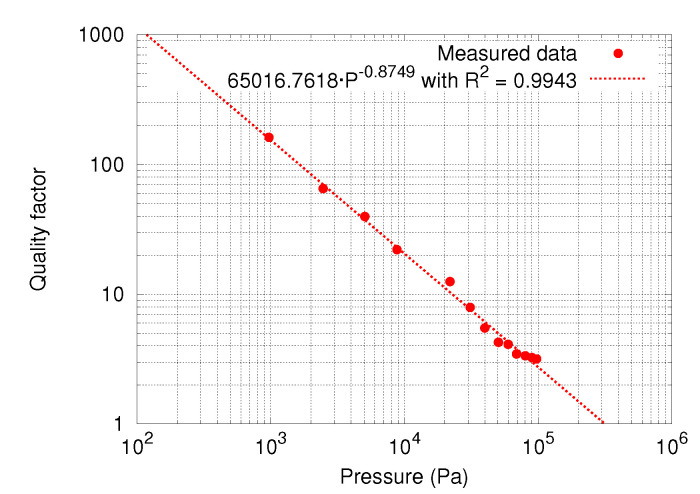
Measured quality factor variation as a function of pressure.

Finally, device quality factor change along pressure variations was also measured and is depicted in Figure 14. Data fitting shows that quality factor changes with pressure as $Q\left(P\right)=65016.76\cdot P^{-0.8749}\;$Pa${}^{-1}$. It is important to characterize this parameter in order to understand how the devices will perform once vacuum packaged: in this situation, the pressure inside a sealed package can no longer be considered to be constant, as it is proportional to temperature [29].

## 6. Discussion

### 6.1. Figures Recalculation

So far, experimental values of capacitance and C-V variation obtained in this article consider all wafer reticles. Unfortunately, during the resonance frequency measurements, some samples in the lower half periphery of the wafer were found collapsed. As a consequence of these non-working devices, the final yield is 85.7%, even though the collapse of these samples is considered to be caused due to wafer handling and not device characteristics, BEOL metals curvature or a faulty vHF release step. For this reason, the real yield is considered, very likely, to be higher. Hence, for the sake of precision, capacitance and C-V variation figures must be recalculated without taking into account the mentioned broken samples. The original and recalculated figures are depicted in Table 1. It can be seen that device capacitance is lower, probably due to a higher average gap, while C-V variation for the resonating samples increase. In both cases, the variability of measurements improves.

### 6.2. BEOL Metal Layers Curvature

**Figure 15:**
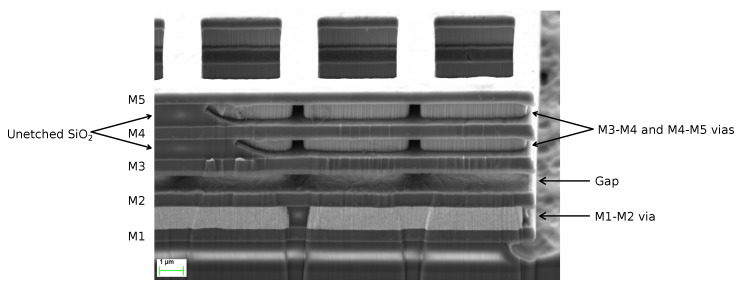
FIB cut of the device plate. The plate periphery is on the right. As it can be seen, acid penetration in between plate metal layers due to the absence of a blocking long via has etched away part of the SiO${}_{2}$. On the left, some oxide remains.

One of the key concerns when designing MEMS sensors using the BEOL metal layers is the curvature of the device structures due to BEOL thin metals residual stress and different temperature coefficients of stacked layers [19,25,26,27,30,31]. The device proposed in this article is not an exception as described in Section 5.2, even though the final device curvature was minimized by using various metal and oxide layers stacked together using long vias, a technique used in numerous CMOS-MEMS devices in the literature [25,26,27]. Unfortunately, due to foundry design rules not allowing the crossing of long vias, it was not possible to put acid penetration blocking vias in the plate periphery, as they would have collided with spring vias. As a consequence, there is some amount of acid penetration between the plate metal layers around the most external part of the plate. Such oxide release is depicted in Figure 15, and it is thought to increase the plate curvature close to plate corners.

One of the issues of single-ended, out-of-plane devices is that variations of the mentioned curvature across the wafer [19] is translated into an important variation of device parameters. A good example can be found in Figure 10a: samples of the upper half wafer periphery have a much higher resonance frequency, which is probably correlated to the higher metal curvature at the wafer periphery observed in Ref. [19] for a similar process. As a result, these samples have a higher quality factor and, thus, a higher sensitivity, yielding to a sensitivity difference when compared with samples in the wafer center. This source of non-uniformity can, in extreme cases, become a source of non-working devices in some parts of the wafer. However, the multi-metal-oxide approach used in the proposed device provides very uniform parameters across most of the wafer. This data, though, must be handled with care, as spacial resolution of measurements is approximately 19.5mm, the distance between wafer samples. Having more and closer samples would probably provide a much smoother Q (and other parameters) change and a higher yield.

### 6.3. Temperature Variations

Temperature changes have an important impact on the spring coefficient as BEOL metals Young’s modulus decreases with temperature. At its turn, the metal only springs’ coefficient soften, which, due to the dependence with resonance frequency $\omega_{r}=\sqrt{k/m}$ make the device resonance to change accordingly [15,16,17]. On the contrary, SiO${}_{2}$ Young’s modulus increases with temperature. Hence, it has been demonstrated that it is possible to minimize temperature dependence of MEMS devices by using metal-oxide stacks [16,26]. Moreover, plate curvature and curvature variation with temperature, have also been observed to improve if plate is designed using multi-layer metal-oxide structures [25,26]. The device proposed in this work was designed following the metal-oxide stack approach in order to perform the current path isolation. As a result the device resonance frequency temperature drift (−413.6ppm$\cdot^{\circ }$C${}^{-1}$, or $-0.041{\%}^{\circ }$C${}^{-1}$) matches very closely the result obtained in the z-axis device of [26]. Avoiding the oxide release at the plate periphery, as explained above, would reduce plate curvature associated with temperature and further minimize temperature drift.

In order to minimize these variations even further, differential devices are usually used, as they compensate temperature and process variations to the first order [32]. Moreover, different gap distances may be compensated, in differential devices, by applying an electrostatic force that minimizes such difference. Some strategies to convert the proposed single-ended device to a differential one are to make it work in the torsional or lateral resonance modes and implement the detection capacitance with fingers in the device sides. However, the most straightforward strategy may be covering the device with a metal layer that, together with the M1 stator and the plate, would make up the differential device. In that case, some precautions must be taken into account. First, rotor to top and bottom stators capacitance must be matched during the design stage. Second, holes must be included in the top electrode in order to allow the acid to penetrate and release the structure. Third, expected rotor curvature must be taken into account in order to avoid the plate to collapse with any of the two stator layers. And fourth, the release step must be carefully performed in order to avoid stiction with the new layer.

## 7. Conclusions

In this article, an out-of-plane, lateral field sensing, 2-axis CMOS-MEMS magnetometer designed with the BEOL metal layers of a standard CMOS process is proposed. Designing the device using such materials, it is possible to manufacture it next to the electronics, side-by-side on the same die, which would allow the further shrink integrated sensing solutions based on MEMS sensors, as well as improving the yield an reducing the manufacturing cost. The device provides complete cancellation of offsets at device level, which is one of the main concerns of Lorentz-force MEMS magnetometers as offset has been reported to reduce dynamic range and worsen long terms instability. The cancellation is performed by placing the current carrying path inside the plate structure, which completely shields the current electrode and insulates it in a Faraday cage-like structure connected to the low impedance *Drive* electrode. The current path isolation has been demonstrated by measuring the capacitance between the current and the sensing electrode. Device integration with the readout electronics, though, is expected to provide further measurements validating such solution. Some device experimental measurements were performed at wafer level, which provides data on the performance of the proposed device across all the wafer. Hence, wafer level measurements of device capacitance, C-V variation, resonance frequency, and quality factor were performed, whose results have been related to previous literature on the topic of BEOL metallization curvatures for similar processes. Concretely, it has been demonstrated that multi-layer stacking techniques previously used to minimize plate curvature provide excellent results when it comes to device parameters scattering. Moreover, sensitivity to magnetic field as a function of pressure has been measured to evaluate the lowest package pressure that would provide the highest sensitivity before driving the device into its non-linear operating region proving that the device is capable to work in the linear regime down to very low pressure. Finally, device resonance frequency temperature dependence was measured, as well as device quality factor variation, which gives an interesting glance on how much device sensitivity drifts with temperature. Such figure is an important parameter to know given the current commercial and automotive demanding temperature specifications.

## Figures and Tables

**Table 1:** Capacitance and C-V variation figures recalculation

	$\bar{C}$ (fF)	$\sigma_{C}$ (fF)	$\Delta C_{C-V}$ (%)	$\sigma_{\Delta C_{C-V}}$ (%)
**All Samples**	118.2	17.7	2.3	1.1
**Only Samples that Resonate**	112.4	10.1	2.7	0.8
